# Examination of sleep in relation to dietary and lifestyle behaviors during Ramadan: A multi-national study using structural equation modeling among 24,500 adults amid COVID-19

**DOI:** 10.3389/fnut.2023.1040355

**Published:** 2023-03-08

**Authors:** Moien A. B. Khan, Ahmed S. BaHammam, Asma Amanatullah, Khaled Obaideen, Teresa Arora, Habiba Ali, Leila Cheikh Ismail, Dana N. Abdelrahim, Mohammed Al-Houqani, Kholoud Allaham, Rand Abdalrazeq, Wahid Sharif Aloweiwi, Somayea Sultana Mim, Ammar Mektebi, Sohrab Amiri, Sahabi Kabir Sulaiman, Syed Fahad Javaid, Mohammad Delwer Hossain Hawlader, Fatimah Isma’il Tsiga-Ahmed, Iffat Elbarazi, Saskiyanto Manggabarani, Gamechu Atomsa Hunde, Sabrina Chelli, Mitra Sotoudeh, MoezAlIslam Ezzat Faris, Ramadan Intermittent Fasting Collaborators, Abasi-Okot Akpan Udoyen

**Affiliations:** National Pirogov Memorial Medical University, Vinnytsia, Ukraine; Faculty of Medicine, Al Quds University, Jerusalem, Palestine; Faculty of Medicine, Helwan University, Cairo, Egypt; Al-Quds University, Bethlehem, Palestine; Faculty of Medicine, Mansoura University Behbbit, Samannoud, Egypt; Qatar University, Mesaieed, Qatar; Department of Family Medicine, College of Medicine and Health Sciences, United Arab Emirates University, Al Ain, United Arab Emirates; RCSI-UCD, Ayer Keroh, Malaysia; Iran Sports Medicine Research Center, Neuroscience Institute Sports Medicine Research Center, Neuroscience Institute Tehran University of Medical Sciences, Tehran, Iran; Kasr Alainy Faculty of medicine, Cairo, Egypt; Kütahya Univerity of Health Sciences, kütahya, Türkiye; Trinity College Dublin, Dublin, Ireland; University of Tunis El Manar, Medical School of Tunis, Military Hospital of Tunis, Tunis, Tunisia; Department Public Health, Universitas Aufa Royhan Di Kota Padangsidimpuan, Padangsidimpuan, Indonesia; Orenburg state Medical University, Orenburg, Russia; Services Institute of Medical Sciences, Services Hospital Lahore House officer Services Institute of Medical Science, Lahore, Paksitan; Lebanese university, Beirut, Lebanon; University of Aleppo, Aleppo, Syria; Chemistry department, American University of Beirut, Beirut, Lebanon; Department of Medicine, Vinnytsia National Medical University, Abuja, Nigeria; Dentistry Programme of Mulawarman University Kerayan, Mulawarman University, Samarinda, Indonesia; Dentistry Programme, Mulawarman University Medical Education, Samarinda, Indonesia; Jordan University of Science and Technology, Irbid, Jordan; Sbks Medical College, Ahmedabad, India; Public Health Asharej, Jawarneh MPH UAEU, Al Ain, United Arab Emirates; Faculty of Medicine, Dental Medicine and Pharmacy of Fez, Sidi Mohammed Ben Abdellah University, Fez, Morocco; College of Medicine, National University for Science and Technology, Seeb, Oman; Ambulatory Healthcare Services, Abu Dhabi, United Arab Emirates; Department of Family Medicine, College of Medicine and Health Sciences, United Arab Emirates University, Abu Dhabi, United Arab Emirates; Dubai Medical College, Dubai, United Arab Emirates; Dubai medical college, Dubai, United Arab Emirates; Ambulatory Healthcare Services, Abu Dhabi, United Arab Emirates; Department of Family Medicine, College of Medicine and Health Sciences, United Arab Emirates University, Abu Dhabi, United Arab Emirates; Department of Endocrinology Syria, Faculty of Medicine, Aleppo University Hospital, University of Aleppo, Aleppo, Syria; Department of surgery, Jaber alahmed hospital, Kuwait City, Kuwait; Spinghar Thoracic Surgery Kabul, Kabul, Afghanistan; College of Food and Agriculture, United Arab Emirates University, Dubai, United Arab Emirates; Alexandria faculty of medicine, General practitioner, Alexandria, Egypt; Department of Nutrition Science, Universitas Muhammadiyah Surakarta, Sukoharjo, Indonesia; Tanjungpura University, Pontianak, Indonesia; MidHudson Family Medicine residency, Institute for family Health Family Medicine, Centerville, United States; OnDokuz Mayis University, Samsun, Turkey; Faculty of Medical Sciences, Lebanese university, Beirut, Lebanon; An-Najah National University, Nablus, Palestine; University of Jordan, Amman, Jordan; Ondokuz Mayis University, Samsun, Turkey; MPH, North South University, Dhaka, Bangladesh; Aljabili Saglik Bilimleri Üniversitesi, Istanbul, Turkey; Monash University, Fawkner, Australia; Ain Shams General Hospital House, Khartoum, Sudan; Tripoli central hospital, Tripoli, Libya; Aleppo University Hospital, Aleppo, Syria; Faculté de Médecine et de Pharmacie de Rabat, Temara, Morocco; NGHA, KAMC, Riyadh, Saudi Arabia; Surgery department (Intern doctor), Princess Basma teaching hospital, Irbid, Jordan; University of Aleppo, Aleppo, Syria; Medical Facuilty, Paktia University, Kabul, Afghanistan; Student Research Committee, Iran Clinical Research Development Center of Imam Khomeini Hospital, Jiroft University of Medical Sciences, Jiroft, Iran; College of Medicine, Qatar University, Doha, Qatar; Faculté de Médecine, de Pharmacie et de Médecine Dentaire de Fès, Fez, Morocco; Kas al aini Clinic, Cairo, Egypt; Department of Physiology, Alzaiem Alazhari University, Khartoum, North Sudan; Department of Human Physiology, Alzaiem Alazhari University, Khartoum, North Sudan; Ministry of Health Internship, Khartoum, Sudan; Ministry of Health, Khartoum, Sudan; The Department of Statistics, The Islamia University of Bahawalpur, Bahawalpur, Pakistan; College of Medicine, Menoufia University, Al Minufiyah, Egypt; Psychology Department of Behavioral Science, Rehman College of Dentistry, Peshawar, Pakistan; CMHS Family Medicine, UAEU, Al Ain, United Arab Emirates; Healthcare Psychology, Abu Dhabi, United Arab Emirates; ICU department, Alexandria Main University Hospital, Alexandria, Egypt; Mosul medical collage, Mosul, Iraq; Ondokuz Mayis University, Samsun, Turkey; Emirates Health Services, Dubai, United Arab Emirates; Faculty of Medicine, Ain Shams University, Cairo, Egypt; Department of Nutrition, Pertamedika College of Health Sciences, Jakarta, Indonesia; School of Nursing and Midwifery, Tehran University of Medical Sciences, Tarbiat Modares University, Tehran, Iran; Shadan Institute of Medical Sciences and Research Centre, Peeramcheru, India; CMH Institute of Medical Sciences, Multan, Pakistan; Birat Medical College and Teaching Hospital Sukhrampur, Krishnanagar, Nepal; Kuwait University, Kuwait City, Kuwait; Jordan University of Science and Technology, Irbid, Jordan; Alexandria Faculty of Medicine, Alexandria, Egypt; College of Medicine, King Saud bin Abdulaziz University for Health Sciences, Riyadh, Saudi Arabia; King Abdulaziz Medical City, Ministry National Guard Health Affairs, Riyadh, Saudi Arabia; King Abdullah International Medical Research Center, Riyadh, Saudi Arabia; Nangarhar Medical Faculty, Timergara, Pakistan; Khatam-Al-Nabieen University, Kabul, Afghanistan; University of Debrecen, Debrecen, Hungary; Faculté de médecine d’Alger, Algiers, Algeria; Sultan Qaboos University (Oman), Seeb, Oman; JSS Medical College, Mysore General Medicine, Kannur, India; Jimma University, Jimma, Ethiopia; DNB GEM Hospital General Medicine, Chennai, India; An-Najah National University, Bethlehem, Palestine; MWACP West African College of Physicians, Federal Neuropsychiatric Hospital, Maiduguri, Nigeria; MBBCh Dubai Medical College, Manama, Bahrain; ^1^College of Medicine and Health Sciences, United Arab Emirates University, Al Ain, United Arab Emirates; ^2^Department of Internal Medicine, College of Medicine, King Saud University, Riyadh, Saudi Arabia; ^3^Knowledge and Research Support Services Department, University of Management and Technology, Lahore, Pakistan; ^4^Sustainable Energy and Power Systems Research Centre, RISE, University of Sharjah, Sharjah, United Arab Emirates; ^5^Department of Psychology, Zayed University, Abu Dhabi, United Arab Emirates; ^6^Department of Clinical Nutrition and Dietetics, College of Health Sciences, University of Sharjah, Sharjah, United Arab Emirates; ^7^Sharjah Institute for Medical and Health Sciences, University of Sharjah, Sharjah, United Arab Emirates; ^8^Internal Medicine College of Medicine and Health Sciences, United Arab Emirates University, Al Ain, United Arab Emirates; ^9^Department of Neurology, Rashid Hospital, Dubai Health Authority, Dubai, United Arab Emirates; ^10^Faculty of Medicine, Mansoura University, Mansoura, Egypt; ^11^School of Medicine, The University of Jordan, Amman, Jordan; ^12^Chattogram International Medical College and Hospital, Chattogram, Bangladesh; ^13^Faculty of Medicine, Kütahya Health Sciences University, Kütahya, Türkiye; ^14^Medicine, Quran and Hadith Research Center, Baqiyatallah University of Medical Sciences, Tehran, Iran; ^15^Department of Internal Medicine, Yobe State University Teaching Hospital, Damaturu, Nigeria; ^16^Department of Psychiatry and Behavioral Science, College of Medicine and Health Sciences, United Arab Emirates University, Al Ain, United Arab Emirates; ^17^Department of Public Health, North South University, Dhaka, Bangladesh; ^18^Department of Community Medicine, Bayero University, Kano, Nigeria; ^19^Institute of Public Health, College of Medicine and Health Sciences, United Arab Emirates University, Al Ain, United Arab Emirates; ^20^Department of Nutrition, Sekolah Tinggi Ilmu Kesehatan Pertamedika, Jakarta, Indonesia; ^21^Faculty of Health Sciences, School of Nursing, Institute of Health, Jimma University, Jimma, Ethiopia; ^22^Royal College of Surgeons in Ireland (Bahrain), Al Muharraq, Bahrain; ^23^Iranshahr University of Medical Sciences, Iranshahr, Iran

**Keywords:** sleep, diet, lifestyle and behavior, fasting, intermittent fasting, Ramadan

## Abstract

**Background:**

Of around 2 billion Muslims worldwide, approximately 1.5 billion observe Ramadan fasting (RF) month. Those that observe RF have diverse cultural, ethnic, social, and economic backgrounds and are distributed over a wide geographical area. Sleep is known to be significantly altered during the month of Ramadan, which has a profound impact on human health. Moreover, sleep is closely connected to dietary and lifestyle behaviors.

**Methods:**

This cross-sectional study collected data using a structured, self-administered electronic questionnaire that was translated into 13 languages and disseminated to Muslim populations across 27 countries. The questionnaire assessed dietary and lifestyle factors as independent variables, and three sleep parameters (quality, duration, and disturbance) as dependent variables. We performed structural equation modeling (SEM) to examine how dietary and lifestyle factors affected these sleep parameters.

**Results:**

In total, 24,541 adults were enrolled in this study. SEM analysis revealed that during RF, optimum sleep duration (7–9 h) was significantly associated with sufficient physical activity (PA) and consuming plant-based proteins. In addition, smoking was significantly associated with greater sleep disturbance and lower sleep quality. Participants that consumed vegetables, fruits, dates, and plant-based proteins reported better sleep quality. Infrequent consumption of delivered food and infrequent screen time were also associated with better sleep quality. Conflicting results were found regarding the impact of dining at home versus dining out on the three sleep parameters.

**Conclusion:**

Increasing the intake of fruits, vegetables, and plant-based proteins are important factors that could help improve healthy sleep for those observing RF. In addition, regular PA and avoiding smoking may contribute to improving sleep during RF.

## Introduction

Muslims constitute the world’s second-largest religious group, as the estimated 2 billion Muslims equates to about 25% of the global population (8 billion) (1). A foundation pillar of Islam is fasting during Ramadan month (2). During this time, adult Muslims are mandated to fast from dawn to sunset. This involves complete abstinence from food, drink, sex, and smoking for 11–20 h per day, depending on the geographical location and the solar season that crosses the lunar fasting month (2). During Ramadan, Muslims take two main meals: the breaking of the day’s fast meal at sunset (“*Iftar*”), and a pre-dawn meal taken in anticipation of the coming fasting hours (“*Suhoor*”) (3). During night hours, Muslims are free to eat, dine, pray, socialize, and perform life activities as permitted by Islamic ruling (4). Fasting during Ramadan is a form of diurnal intermittent fasting or a time-restricted eating model (5), with dietary and lifestyle changes that persist for 29–30 consecutive days around fixed time points.

Various forms of intermittent fasting have been reported to induce a plethora of health benefits (6, 7). Among these, religious forms of fasting such as Ramadan intermittent fasting (RF) have received continuous attention (7). For example, several beneficial health impacts of RF have been demonstrated in numerous studies over the last seven decades (8). Previous studies reported RF was associated with reduced metabolic syndrome components (9), reduced body weight (10), reduced body fatness (11) with emphasis on visceral fats (12), decreased inflammatory and oxidative stress markers (13), and improved glucometabolic regulation (14) and liver function tests (15). On the other hand, RF has also been associated with disrupted circadian rhythms, and changes to sleep-wake timings as well as hormones such as leptin, adiponectin, ghrelin, cortisol, and melatonin (16–19). These changes were associated with sudden changes in meal timings, diet composition, food group consumption, and sleep continuity during Ramadan, which may interfere with the metabolic impacts of RF (18, 20, 21).

Insufficient sleep has previously been associated with weight gain and cardiometabolic risk (22). Of note, several studies reported significant and sudden delays in bedtime and waking time and a considerable reduction in total sleep time during RF (20, 23), which may contribute to weight gain but be counteracted by reduced food intake during this time. Empirical results for non-RF times showed that diet quality, timing, and quantity impacted sleep duration and sleep quality (24, 25). Therefore, it is relevant to assess the association between sleep and dietary and lifestyle factors during RF. However, Ramadan-related dietary and lifestyle practices vary across nations and cultures, which may impact sleep length, sleep-wake timings, and sleep disruption to different degrees. This means results for sleep and lifestyle changes obtained from a specific country or culture cannot be generalized to other countries. This is bolstered by the data that dietary, sleep, and lifestyle behaviors vary substantially across the world’s population and, as a result, so does metabolic health status (26–34).

The present study examined changes in dietary and lifestyle factors during RF and explored their associations with sleep duration and quality among fasting people from a range of different cultures and ethnicities. This study was based on the hypothesis that RF is associated with significant changes in sleep timing, duration, and quality. We also hypothesized that changes in sleep quality and duration are likely to be affected by various dietary and lifestyle changes that occur during the month of fasting. Because multiple lifestyle factors are closely connected with sleep outcomes during RF, we used structural equation modeling (SEM) to examine how specific dietary and lifestyle modifications influenced three sleep parameters (sleep duration, sleep quality, and sleep disturbances) among fasting Muslims during RF in several countries with varying dietary and lifestyle habits and background cultures.

## Materials and methods

### Study design, settings, sample size, and population

The present study used a cross-sectional, observational design with targeted recruitment of adult Muslims who fasted during Ramadan month. This study obtained data related to dietary and lifestyle changes during RF in the context of the COVID-19 pandemic. Data collection started on May 10, 2021 (corresponding to the 27th Ramadan month in 1,442 Hijri) and concluded on 10 June 2021 (29th *Shawwal* 1,442 Hijri). The inclusion criterion was adult Muslims (aged ≥18 years) who observed RF. We excluded those who answered “Yes” to the question “Have you been diagnosed with or treated for a mental health problem?” Individuals following specific diets and those engaged in shift work were also asked not to complete the survey at the beginning of the electronic questionnaire.

This study was initiated and supervised by researchers in the UAE. Snowball sampling was used to collect data. Voluntary collaborators from various countries were invited through Facebook research groups. In total, 116 collaborators from 29 countries participated in data collection. These research collaborators randomly drew on their networks using the different web-based platforms such as emails, WhatsApp, and Facebook. Each researcher was given a survey form along with a unique link to collect data, and data were pooled when data collection was completed. This method facilitated the wide distribution of the survey questionnaire during the pandemic period when there were many lockdown restrictions. An *a priori* G* power estimate was calculated for each country using one-tailed Student’s *t*-tests and a bivariate correlation analysis model to identify the required sample size. An estimated effect size of 0.2, alpha error of 0.05, and power of 0.90 indicated that a minimum of 207 participants were needed from each country included in this study.

This study adhered to the code of ethics as set out in the Declaration of Helsinki (35). Before data collection started, approval was obtained from the Social Sciences Research Ethics Committee of the United Arab Emirates University (Approval number ERS-2021-7308) and Tehran University of Medical Science (Approval number IR.TUMS.FNM.REC.1400.022). Furthermore, participants were informed about the objectives and procedures of the study before providing informed consent. No monetary or non-monetary incentive was given for participation. Participants gave their consent as a first step on the online questionnaire form.

### Tool development and translation

The data collection tool was prepared as a structured, self-administered, electronic questionnaire that assessed demographic information, dietary intake, eating habits, sleep parameters, and physical activity (PA) level during Ramadan. Web-based questionnaires were circulated, and participants were recruited using convenience and snowball sampling methods. As no changes to the questionnaire were deemed necessary after the pilot testing, participants from the pilot study samples were included in the final sample for the analyses. The questionnaire developed for this study was translated into 13 different languages (Arabic, English, French, Turkish, Urdu, Bengali, Persian, Indonesian, Pashto, Dari, Amharic, Malay, and Afaan Oromo). The questionnaire translation and cultural adaptation process followed the “Principles of Good Practice for the Translation and Cultural Adaptation Process” (36). First, forward translations were performed by two independent translators fluent in both English and the local language, and then back-translated to English. After reviewing the backward translations, the version of the questionnaire for each language was proofread and underwent further editing (as necessary) before being pilot tested with 30 participants, which resulted in the final versions of the survey questionnaire (36). A structured, self-administered questionnaire was developed from previously validated questionnaires (37–43). After collecting relevant questions from these questionnaires, the self-structured questionnaire for this study was translated and then pre-tested to ensure the questions were unambiguous.

### Data cleaning

Response options with the same meaning were unified for all variables. Participants who missed answering major questions (e.g., consent, age, sex, nationality, education, occupation, health status estimation, country of residence, and the number of fasting days) were omitted from the analyses. Those that did not fast during Ramadan were also omitted; participants that reported “zero” as the number of fasting days were considered non-fasting and omitted from our study. Body mass index (BMI, kg/m^2^) was calculated for all participants using their reported height and weight, after which they were classified and analyzed using established BMI categories (43). Participant’s country of residence was used to cluster the study population into three main geographical regions rather than their nationality. These regions were based on the latest update of the world map. Participants from non-countries (e.g., territories and self-proclaimed countries) were omitted. Dietary pattern questions were re-classified from four category variables into two category variables. Finally, we excluded questionnaires for participants younger than 18 years and those with abnormal inserted values (weight, height, age).

### Questionnaire measures

Sociodemographic information collected included sex and age (years), country of residence, nationality, region, marital status (single, married, divorced, or widowed), living area (city, town, or village), household income, living conditions (alone, with friends, or with family), education level, and the number of fasting days experienced (this question reflected the fact that some people may fast less than 29–30 days for various reasons such as travel or illness).

As this was a multicenter study, total household income was classified into five quintiles for standardization purposes: upper (top 20%), upper-middle (upper 20%), basic middle (middle 20%), marginal middle (lower 20%), and lower (lowest 20%) (44). In addition, participants were asked to classify their economic status as per their economic conditions in relation to their community and local region/country. A smoking behavior questionnaire was used to identify smoking behaviors (cigarette and shisha) before and during the month of Ramadan (37, 38).

Questions assessing participants’ PA levels were derived from the International Physical Activity Questionnaire Short Form (41). We assessed participants’ general PA levels, as well as the frequency of 10 min of heavy and light PA, and overall self-evaluated energy levels before and during RF. We merged heavy and light PA into a single PA variable for analysis. Questions covering screen time and sleep parameters were based on the validated Copenhagen Psychosocial Questionnaire (41, 42). Participants provided information on time spent using computers for work as well as television/social media or computers for entertainment (both day and nighttime) and spending time with family. Self-reported computer/laptop use for study, work, or entertainment was merged into one variable. We collected participants’ self-reported height (cm) and weight (kg) and then calculated and categorized BMI based on the World Health Organization definition (43).

The dietary part of the questionnaire collected information on several factors before and during RF, including modification of eating practices, snacking frequency, intake of water, consuming large quantities of food, feeling hungry, and the consumption of 20 different food items and beverages (vegetables, fruits, cereals, oils and fats, milk and milk products, pulses/dried legumes, dates, fish and seafood, white meat, red meat, sugar, salt, fried foods, salty snacks, carbonated beverages, energy drinks, tea and coffee, bakery products, homemade foods, traditional foods, delivered food, restaurant food, and snacks) (39, 40). To evaluate dietary intake, we classified foods into eight groups: (I) fruits, vegetables, and dates; (II) cereals, pulses (dried legumes), and pastries; (III) milk products, fish and seafood, white meat (chicken and turkey), and red meat; (IV) oils, fats, and fried foods; (V) sugar, carbonated beverages, energy drinks, and tea and coffee; (VI) salt and salty snacks; (VII) homemade food; and (VIII) traditional foods. Each of these food item groups had four response options in the original questionnaire: “not consumed,” “decreased,” “remained as usual,” or “increased.” For the analyses, these responses were re-categorized into binary categories using two different couple of terms based on the type of food item, first couple of term was sufficient/insufficient, which was defined as sufficient (remained as usual or increased intake), or insufficient (no intake or decreased intake). These terms were applied for (I) fruits, vegetables, and dates; (II) cereals, pulses (dried legumes), and pastries; (III) milk products, fish and seafood, white meat (chicken and turkey), and red meat. A second couple of terms is frequent/infrequent, where frequent was denoted (remained as usual or increased intake) while infrequent (no intake or decreased intake). These terms were used for (IV) oils, fats, and fried foods; (V) sugar, carbonated beverages, energy drinks, and tea and coffee; (VI) salt and salty snacks; (VII) homemade food; and traditional foods, because there was no limit of the sufficiency of these four groups. Similarly, some other variables had four response options also in the original questionnaire: “not consumed,” “decreased,” “remained as usual,” or “increased.” Those variables were categorized in the same way into binary variables as frequent/infrequent style, where frequent was denoted (remained as usual or increased intake) while infrequent (no intake or decreased intake). This was applied also to the following behaviors: consuming water, practicing physical activity, consumption of delivered food, restaurant dining, and using the computer. While smoking was originally defined as smokers and non-smokers.

### Sleep parameters

Three sleep parameters were assessed based on participants’ estimations before and during RF: sleep quality, sleep duration (<7, 7–9, and >9 h), and sleep disturbance. We considered 7–9 h of sleep per night as optimal sleep duration (45), <7 h as short sleep duration, and >9 h as long sleep duration, based on previously published consensus by sleep experts (46). Sleep quality was self-reported as poor, good, or very good. Participants were also asked to indicate if they experienced any of the listed sleep disturbances before and during RF: (I) slept poorly and restlessly; (II) hard to go to sleep; (III) woke too early and was unable to get back to sleep; (IV) woke several times and found it difficult to get back to sleep; and (V) no sleep disturbances. These questions were drawn from a reliable and validated instrument (42).

### Statistical analyses

Statistical analyses and the application of SEM were based on the consideration of dietary and lifestyle behaviors as the main exposures for people observing RF, and the three sleep quality parameters (sleep duration, perceived sleep quality, and sleep disturbance) as the main outcomes of interest.

### Structural equation modeling

Structural equation modeling is a multivariate statistical analysis technique used to analyze structural relationships. This technique combines multiple regression and factor analyses and is used to investigate the structural relationship between measured variables and latent constructs. This technique allows accurate estimations of interrelated variables and multiple dependencies in a single analysis (47, 48). Two types of variables are used in SEM: exogenous and endogenous variables. Exogenous variables are equivalent to independent variables, and endogenous variables are equivalent to dependent variables. In this study, SEM was performed using Smart PLS 3 software (49). Dietary intake and eating and lifestyle behaviors (including consumption of major food groups, delivered food, dining in restaurants, smoking, PA, and computer use) were considered exogenous variables, and sleep duration, sleep quality, and sleep disturbance were considered endogenous variables.

Evaluation of the model was performed in two steps. First, we evaluated the measurement model to assess the psychometric properties of the variables. In the second step, the structural model was evaluated by considering multicollinearity, multiple correlations, predictive relevance, and path coefficients. In the model examined in the present study, only discriminant validity was calculated. As we used observed variables, reliability and convergent validity for variables with a single indicator should equal 1. Furthermore, we assessed the correlation between independent variables in the model to explore multicollinearity issues. The predictive relevance (*Q*^2^) of the model was calculated by eliminating specific data points. The final step in the evaluation of the structural model was to identify significant paths or associations between independent and dependent variables.

Discriminant validity was measured using the Heterotrait-Heteromethod ratio of correlations (HTMT) criteria, which is an advanced method of determining discriminant validity. HTMT represents the ratio of Heterotrait-Heteromethod correlations to Monotrait-Heteromethod correlations. HTMT correlations reflect the correlation of indicators with other constructs in the model, whereas Monotrait-Heteromethod correlations are the correlations of indicators with the same constructs in a model (50). An HTMT value between two constructs below 0.9 suggests that discriminant validity has been established and the variables are distinct from each other (51). Good discriminant validity means that indicators of all variables are distinct from each other (Supplementary Table 1).

The structural model was then evaluated by exploring multicollinearity, multiple correlations, and the predictive relevance of the model. The correlation between independent variables was assessed to explore multicollinearity issues using variance inflation factor (VIF) statistics. Hair et al. (52) indicated the VIF value should be less than 5. We found that the VIF values for the independent variables corresponding to all three sleep parameters were <5.0; therefore, multicollinearity was not present. The predictive capabilities of the model were calculated using the coefficient of determination (*R*^2^) and predictive relevance (*Q*^2^). *R*^2^ was obtained using a bootstrapping procedure with 5,000 sub-samples, calculated through the blindfolding procedure by selecting an omission distance of 7.

A descriptive table was prepared using SPSS version 26 (IBM, Armonk, NY, USA). Categorical variables were expressed as frequency and percentage. Continuous data were described using mean ± standard deviation. Descriptive statistics were used to present sociodemographic data. STATA version 16.1 (StataCorp^®^ LLC, TX, USA) was used for the statistical analyses. Any missing data were predicted using linear regression. *P*-values < 0.05 were considered statistically significant.

## Results

**FIGURE 1:**
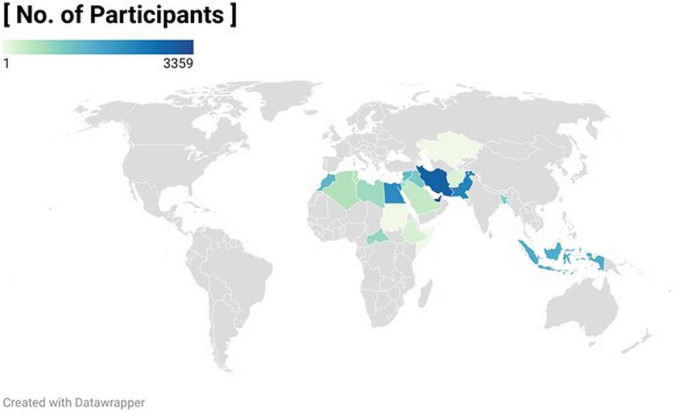
Worldmap showing the number of participants using color density for the 27 participating countries.

In total, 28,179 participants were recruited for this study and provided data; 3,638 participants were excluded after applying the exclusion criteria, leaving 24,541 participants for inclusion in the final analyses. Data were collected from 27 countries distributed across three main regions (Figure 1). The Middle East and North Africa (MENA) region, including The Gulf Cooperation Council (GCC) countries (Bahrain, n = 688; Kingdom of Saudi Arabia, *n* = 421; Qatar, *n* = 466; and the UAE, *n* = 3,359); Middle East non-GCC countries (Egypt, *n* = 2171; Iraq, *n* = 1,101; Iran, *n* = 2,946; Jordan, *n* = 680; Palestine, *n* = 1,706; Syria, *n* = 1,356; and Yemen, *n* = 845); and North Africa countries (Algeria, *n* = 592; Ethiopia, *n* = 212; Libya, *n* = 777; Morocco, *n* = 1,390; and Tunisia, *n* = 529). The South Asian region included Bangladesh (*n* = 12,69) and Pakistan (*n* = 2,312). The Southeast Asia region comprised Indonesia (*n* = 1,605).

**FIGURE 2:**
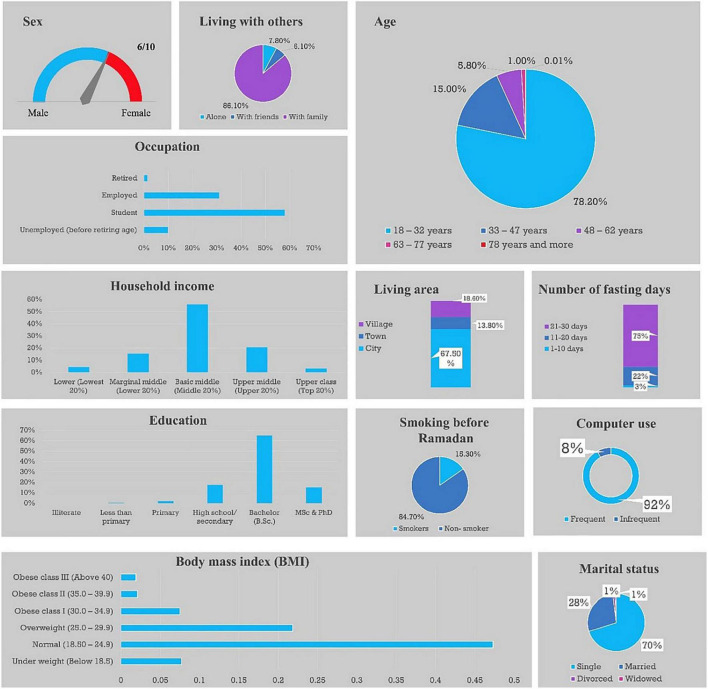
Sociodemographic characteristics of the 24,500 study participants.

The MENA region with its three sub-regions (GCC, Middle Eastern non-GCC, and North Africa) comprised the largest proportion of the study population, followed by the South Asian region and the Southeast Asia region (Table 1). As depicted in Figure 2, females comprised almost two-thirds of the study participants. The most common age group was 18–32 years, followed by 33–47 years. The majority of participants were single and about 28% were married. The largest proportion of participants lived in cities, followed by villages and towns. Based on the participant’s country of residence, the majority of participants had basic middle-income status, followed by the upper-middle and marginal middle. The largest proportion of participants was living with their families and engaged in fasting for most of Ramadan month (21–30 days), followed by those fasting for two-thirds and one-third of the fasting month. Most participants were non-smokers, and around two-thirds had a university-level education. More than half of the participants were students, and one-third were employed. Medication in computer use was common among participants during Ramadan. Finally, the largest proportion of participants had normal body weight (47.3%), followed by those with overweight, underweight, and class I obesity (Figure 2). Detailed sociodemographic characteristics for the three regions are reported in Table 2.

**TABLE 1:** Sociodemographic characteristics of the study population (*N* = 24,541).

Sociodemographic variable	MENA region (*n* = 19,355) *n* (%)	South Asian region (*n* = 3,580) *n* (%)	Southeast Asia region (*n* = 1,606) *n* (%)
**Sex**			
Male	7,075 (36.6)	1,766 (49.3)	919 (57.2)
Female	12,280 (63.4)	1,814 (50.7)	687 (42.8)
**Age (years)**			
18–32	15,341 (79.3)	2,994 (83.6)	859 (53.5)
33–47	2,970 (15.3)	449 (12.5)	253 (15.8)
48–62	930 (4.8)	115 (3.2)	382 (23.8)
63–77	108 (0.6)	19 (0.5)	108 (6.7)
≥ 78	2 (0.01)	3 (0.1)	4 (0.2)
**Marital status**			
Single	13,772 (71.2)	2,687 (75.1)	754 (46.9)
Married	5,206 (26.9)	840 (23.5)	810 (50.4)
Divorced	258 (1.3)	31 (0.9)	22 (1.4)
Widowed	119 (0.6)	22 (0.6)	20 (1.2)
**Living area**			
City	14,011 (72.4)	2,045 (57.1)	511 (31.8)
Town	2,217 (11.5)	713 (19.9)	466 (29.0)
Village	3,124 (16.1)	822 (23.0)	629 (39.2)
**Household income**			
Lower (lowest 20%)	882 (4.6)	136 (3.8)	98 (6.1)
Marginal middle (lower 20%)	2,945 (15.2)	438 (12.2)	410 (25.5)
Basic middle (middle 20%)	10,920 (56.4)	2,082 (58.2)	745 (46.4)
Upper middle (upper 20%)	3,961 (20.5)	836 (23.4)	294 (18.3)
Upper (top 20%)	647 (3.3)	88 (2.5)	59 (3.7)
**Living with others**			
Alone	1,532 (7.9)	255 (7.1)	128 (8.0)
With friends	1,101 (5.7)	266 (7.4)	135 (8.4)
With family	16,722 (86.4)	3,059 (85.4)	1,343 (83.6)
**Number of fasting days**			
1–10	444 (2.3)	86 (2.4)	95 (5.9)
11–20	4,276 (22.1)	754 (21.1)	447 (27.8)
21–30	14,635 (75.6)	2,740 (76.5)	1,064 (66.3)
**Smoking status**			
Smoker	3132 (16.2)	364 (10.2)	247 (15.4)
Non-smoker	16,222 (83.8)	3,216 (89.8)	1359 (84.6)
**Education**			
Illiterate	3 (0.01)	2 (0.1)	–
Less than primary	64 (0.3)	23 (0.6)	62 (3.9)
Primary	218 (1.1)	29 (0.8)	258 (16.1)
High school/secondary	3,451 (17.8)	539 (15.1)	287 (17.9)
Bachelor’s	12,734 (65.8)	2,368 (66.1)	807 (50.2)
Master’s or doctorate	2,880 (14.9)	618 (17.2)	192 (12.0)
**Occupation**			
Unemployed (pre-retirement age)	1,798 (9.3)	260 (7.3)	343 (21.4)
Student	11,202 (57.9)	2,409 (67.3)	607 (37.8)
Employed	6,095 (31.5)	875 (24.4)	614 (38.2)
Retired	260 (1.3)	36 (1.0)	42 (2.6)
**Computer use**			
Increased	17,933 (92.7)	3,147 (87.9)	1,473 (91.7)
Decreased	1422 (7.3)	433 (12.1)	133 (8.3)
**Body mass index (kg/m^2^)**	**(*n* = 17,972)**	**(*n* = 2,923)**	**(*n* = 790)**
Underweight (<18.5)	1,539 (8.0)	318 (8.9)	40 (2.5)
Normal (18.50–24.9)	9,785 (50.6)	1,431 (40.0)	391 (24.3)
Overweight (25.0–29.9)	4,427 (22.9)	682 (19.1)	243 (15.1)
Obese class I (30.0–34.9)	1,508 (7.8)	248 (6.9)	74 (4.6)
Obese class II (35.0–39.9)	409 (2.1)	98 (2.7)	19 (1.2)
Obese class III (≥40)	304 (1.6)	146 (4.1)	23 (1.4)

**TABLE 2:** Collinearity statistics (variance inflation factors) for the study variables (*N* = 24,541).

Eating, dietary, and lifestyle behavior	Sleep duration	Self-described sleep quality	Sleep disturbance
Smoking	1.02	1.02	1.02
Frequency of ordered food delivery	1.24	1.24	1.24
Frequency of eating out in restaurants	1.23	1.23	1.23
Frequency of computer use	1.03	1.03	1.03
Physical activity	1.02	1.02	1.02
Eating vegetables, fruits, dates	1.19	1.19	1.19
Cereals, pulses (dried legumes), bakery products consumption (Plant-protein sources)	1.30	1.30	1.30
Milk, fish, chicken, and meat consumption (Animal-protein sources)	1.27	1.27	1.27
Oils, fats, fried food consumption	1.35	1.35	1.35
Sugars, carbonated beverages, energy drinks, coffee, and tea consumption (caffeine sources)	1.32	1.32	1.32
Adding salt, and salty snacks consumption	1.41	1.41	1.41
Eating homemade, traditional foods	1.27	1.27	1.27

**FIGURE 3:**
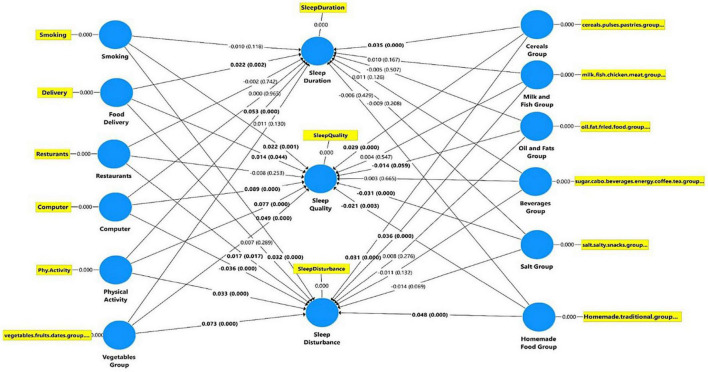
Structural equation modeling (SEM) for the relationships between sleep components and the dietary and lifestyle behaviors during Ramadan fasting month among the 24,500 study participants.

The SEM output is presented in Figure 3, which depicts the relationships between the three sleep parameters and various dietary and lifestyle behaviors practiced during RF. Collinearity statistics (VIF) of the study variables are reported in Table 2. As shown in Table 2, all independent variables (eating behaviors, dietary intake, and lifestyle behaviors) were weak but significant predictors of the dependent variables (three sleep quality parameters). The coefficient of determination (*R*^2^) and predictive relevance (*Q*^2^) of the model are reported in Table 3.

**TABLE 3:** Coefficient of determination (*R*^2^) and predictive relevance (*Q*^2^) of the model (*N* = 24,541).

	*R* ^2^	*Q* ^2^
Sleep duration	0.01***	0.001
Self-evaluated sleep quality	0.02***	0.02
Sleep disturbance	0.02***	0.02

Highly significant, ***p < 0.001.

Table 4 shows that people who modified their delivered food pattern and consumed sufficient cereals, pulses/dried legumes, and bakery products (plant-based proteins) during RF reported optimal sleep duration (7–9 h). Furthermore, sufficient PA was positively associated with optimal sleep duration. Apart from the positive effects of consumption of delivered foods, the impact of sufficient consumption of plant-based proteins and sufficient PA on sleep duration had a weak effect size.

**TABLE 4:** Associations between dietary and lifestyle behavior modifications and reported sleep duration (*N* = 24,541).

Eating, dietary, and lifestyle behavior	B	SE	P	Confidence intervals		*f* ^2^
Smoking	-0.01	0.01	0.12	–0.02	0.001	0.001
Frequent ordering of food delivery	0.02	0.01	0.001	0.01	0.04	0.001
Frequent eating out in restaurants	0.001	0.01	0.75	–0.02	0.01	0.001
Frequent computer use	0.001	0.01	0.96	–0.01	0.01	0.001
Physical activity	0.05	0.01	0.001	0.04	0.07	0.001
Eating vegetables, fruits, dates	0.01	0.01	0.12	0.001	0.02	0.001
Cereals, pulses (dried legumes), bakery products consumption (Plant-protein sources)	0.04	0.01	0.001	0.02	0.05	0.001
Milk, fish, chicken, meat consumption (Animal-protein sources)	0.01	0.01	0.17	0.001	0.02	0.001
Oils, fats, fried food consumption	0.001	0.01	0.51	–0.02	0.01	0.001
Sugars, carbonated beverages, energy drinks, coffee, and tea consumption (caffeine sources)	0.01	0.01	0.13	0.001	0.03	0.001
Adding salt, and salty snacks consumption	-0.01	0.01	0.22	–0.02	0.01	0.001
Eating homemade, traditional foods	-0.01	0.01	0.42	–0.02	0.01	0.001

SE, standard error; CI, confidence interval. Sleep duration (0=poor, 1=good); smoking status (0=smoker, 1=non-smoker); frequency of delivered food (0=frequent, 1=infrequent); frequency of restaurant dining (0=frequent, 1=infrequent); frequency of computer use (0=frequent, 1=infrequent); physical activity (0=insufficient, 1=sufficient); vegetables, fruits, and dates consumption (0=insufficient, 1=sufficient); cereals, pulses, and bakery products consumption (plant-protein sources) (0=insufficient, 1=sufficient); milk, fish, chicken, and meat consumption (animal-protein sources) (0=insufficient, 1=sufficient); oils, fats, and fried food consumption (0=insufficient, 1=sufficient), sugar, carbonated beverages, energy drinks, coffee, and tea consumption (0=insufficient, 1=sufficient); adding salt, and salty snacks consumption (0=insufficient, 1=sufficient); and eating homemade traditional foods (0=insufficient, 1=sufficient).

Table 5 shows that smoking was associated with lower self-evaluated sleep quality. Furthermore, sufficient PA and sufficient consumption of vegetables, fruits, dates, cereals, pulses, and bakery products during RF were associated with better subjective sleep quality. Participants’ quality of sleep improved when they reduced their consumption of delivered food during Ramadan. However, consumption of salt and salty snacks and eating homemade traditional foods was associated with lower sleep quality, although the effect size of the impact of these factors on sleep quality was weak. Smoking was associated with increased sleep disturbance during RF (Table 6). Both modifications in dining out and eating homemade traditional foods were positively associated with sleep disturbance. Unexpectedly, sufficient PA, sufficient consumption of fruits and vegetables, and sufficient consumption of both plant and animal-based proteins were positively associated with sleep disturbance; the effect size for these factors was weak but significant. At the regional levels, significant differences were found between the three regions concerning the various dietary, sleep, and lifestyle behaviors, except for the delivery of food delivery (Table 7).

**TABLE 5:** Associations between dietary and lifestyle behavior modification and self-evaluated sleep quality (*N* = 24,541).

Eating, dietary, and lifestyle behavior	B	SE	P	Confidence intervals		*f* ^2^
Smoking	-0.01	0.01	0.12	–0.02	0.001	0.001
Frequent ordering of food delivery	0.02	0.01	0.001	0.01	0.04	0.001
Frequent eating out in restaurants	0.001	0.01	0.75	–0.02	0.01	0.001
Frequent computer use	0.001	0.01	0.96	–0.01	0.01	0.001
Physical activity	0.05	0.01	0.001	0.04	0.07	0.001
Eating vegetables, fruits, dates	0.01	0.01	0.12	0.001	0.02	0.001
Cereals, pulses (dried legumes), bakery products consumption (Plant-protein sources)	0.04	0.01	0.001	0.02	0.05	0.001
Milk, fish, chicken, meat consumption (Animal-protein sources)	0.01	0.01	0.17	0.001	0.02	0.001
Oils, fats, fried food consumption	0.001	0.01	0.51	–0.02	0.01	0.001
Sugars, carbonated beverages, energy drinks, coffee, and tea consumption (caffeine sources)	0.01	0.01	0.13	0.001	0.03	0.001
Adding salt, and salty snacks consumption	-0.01	0.01	0.22	–0.02	0.01	0.001
Eating homemade, traditional foods	-0.01	0.01	0.42	–0.02	0.01	0.001

SE, standard error; CI, confidence interval. Sleep duration (0=poor, 1=good); smoking status (0=smoker, 1=non-smoker); frequency of delivered food (0=frequent, 1=infrequent); frequency of restaurant dining (0=frequent, 1=infrequent); frequency of computer use (0=frequent, 1=infrequent); physical activity (0=insufficient, 1=sufficient); vegetables, fruits, and dates consumption (0=insufficient, 1=sufficient); cereals, pulses, and bakery products consumption (plant-protein sources) (0=insufficient, 1=sufficient); milk, fish, chicken, and meat consumption (animal-protein sources) (0=insufficient, 1=sufficient); oils, fats, and fried food consumption (0=insufficient, 1=sufficient), sugar, carbonated beverages, energy drinks, coffee, and tea consumption (0=insufficient, 1=sufficient); adding salt, and salty snacks consumption (0=insufficient, 1=sufficient); and eating homemade traditional foods (0=insufficient, 1=sufficient).

**TABLE 6:** Associations between dietary and lifestyle behaviors and sleep disturbance (*N* = 24,541).

Eating, dietary, and lifestyle behavior	B	SE	P	Confidence intervals		*f* ^2^
Smoking	0.03	0.01	0.001	0.02	0.04	0.001
Frequent ordering of food delivery	0.01	0.01	0.29	–0.01	0.02	0.001
Frequent eating out in restaurants	0.02	0.01	0.02	0.001	0.03	0.001
Frequent computer use	-0.04	0.01	0.001	–0.05	–0.02	0.001
Physical activity	0.03	0.01	0.001	0.02	0.05	0.001
Eating vegetables, fruits, dates	0.07	0.01	0.001	0.06	0.09	0.001
Cereals, pulses, bakery products (Plant-protein sources)	0.03	0.01	0.001	0.02	0.04	0.001
Milk, fish, chicken, meat consumption (Animal-protein sources)	0.04	0.01	0.00	0.02	0.05	0.001
Oils, fats, fried food consumption	-0.01	0.01	0.28	–0.02	0.01	0.001
Sugars, carbonated beverages, energy drinks, coffee, and tea consumption (caffeine sources)	-0.01	0.01	0.13	–0.03	0.001	0.001
Adding salt, and salty snacks consumption	-0.01	0.01	0.07	–0.03	0.001	0.001
Eating homemade, traditional foods	0.05	0.01	0.001	0.03	0.06	0.001

SE, standard error; CI, confidence interval. Sleep duration (0=poor, 1=good); smoking status (0=smoker, 1=non-smoker); frequency of delivered food (0=frequent, 1=infrequent); frequency of restaurant dining (0=frequent, 1=infrequent); frequency of computer use (0=frequent, 1=infrequent); physical activity (0=insufficient, 1=sufficient); vegetables, fruits, and dates consumption (0=insufficient, 1=sufficient); cereals, pulses, and bakery products consumption (plant-protein sources) (0=insufficient, 1=sufficient); milk, fish, chicken, and meat consumption (animal-protein sources) (0=insufficient, 1=sufficient); oils, fats, and fried food consumption (0=insufficient, 1=sufficient), sugar, carbonated beverages, energy drinks, coffee, and tea consumption (0=insufficient, 1=sufficient); adding salt, and salty snacks consumption (0=insufficient, 1=sufficient); and eating homemade traditional foods (0= infrequent, 1= frequent).

**TABLE 7:** Comparison between the three regions (during Ramadan).

Dietary and lifestyle behaviors (Bad behaviors)	MENA region	South Asian region	Southeast Asia region	*P*-value
% Of smokers	85.6%^a^	91.7%^b^	88.0%^c^	0.001*
% Of frequently delivered food consumption	1.8%	2.2%	2.4%	0.107
% Of frequent restaurant dining	1.1%^a^	1.1%^a^	1.9%^b^	0.009*
% Of frequent computer use	91.4%^a^	86.1%^b^	75.9%^c^	0.001*
% Of insufficient physical activity	86.2%^a^	83.7%^a^	74.5%^b^	0.001*
% Of insufficient vegetables, fruits, and dates consumption	20.2%^a^	15.3%^b^	13.8%^b^	0.001*
% Of insufficient cereals, pulses (dried legumes), and bakery products consumption (plant-protein sources)	32.0%^a^	37.1%^b^	27.0%^c^	0.001*
% Of insufficient milk, fish, chicken, and meat consumption (animal-protein sources) consumption	19.1%^a^	20.9% ^b^	14.2% ^c^	0.001*
% Of frequent oils, fats, and fried food consumption	77.7% ^a^	83.3%^b^	86.6%^b^	0.001*
% Of frequent sugar, carbonated beverages, energy drinks, coffee, and tea consumption (caffeine sources)	55.5%^a^	64.5%^b^	70.5%^c^	0.001*
% Of frequent adding salt, and salty snacks consumption	77.3%^a^	81.6%^b^	85.0%^c^	0.001*
% Of insufficient eating homemade traditional foods	11.2%^a^	9.7%^b^	9.8%^ab^	0.011*

Significant, *p < 0.05. Different letters denote a significant change.

## Discussion

This large-scale study examined the relationship between dietary and lifestyle factors and sleep duration and quality during RF across various countries in the context of the COVID-19 pandemic. Our hypothesis that RF was associated with significant changes in sleep timing, duration, and quality was confirmed. Furthermore, our hypothesis that changes in sleep quality and duration were related to changes in dietary and lifestyle behaviors during RF was also confirmed. In line with the observed differences in the different dietary, sleep, and lifestyle behaviors between the investigated regions, changes in these outcomes were significantly different between the three investigated regions upon Ramadan, except for the delivery of food. This could be understood in light of the basic inherent differences in cultural, economic, and ethnic fundamental backgrounds between the different regions.

## Sleep quality components

### Sleep duration

We found a conflicting relationship between the three sleep quality parameters and PA, dietary intake, and food group consumption both at home and dining out; this may be attributable to the presence of various undetected confounding factors. Such discrepancies are expected when using an observational research design. This study revealed that sleep duration, self-evaluated sleep quality, and sleep disturbance had variable associations with the investigated dietary and lifestyle factors before and during RF. Reduced consumption of delivered foods, sufficient consumption of plant-based proteins, and sufficient PA were all associated with better-self-evaluated sleep quality in our study population. Food quantity, quality, and eating habits along with PA are considered major determinant factors that profoundly affect sleep quality (24, 25, 30, 53). Delivered foods are typically takeaway fast food, which tends to be energy-dense and high in fat, sugar, and animal protein, and low in plant-based and dietary fiber foods (54–56). Previous studies reported that excessive and reduced consumption of these energy-dense foods was associated with increased body weight and adverse health consequences, such as poor sleep quality and short sleep duration (57, 58). We found an unexpected positive association between sleep duration and the decreased modification in the consumption of delivered foods. This may imply the presence of confounders and other undetected interfering factors in the relationship between sleep duration and consumption of delivered foods. Further, this unexpected positive correlation between sleep duration with the frequent ordering of delivered food could be due to the Covid-19 situation where the individuals are under-locked down or in mobility-restricted situations and cannot eat out.

Unique plant proteins and bioactive phytochemicals mean that plant-based food has a high anti-inflammatory potential that maintains good health, lowers the risk for chronic ailments (59), and improves sleep quality (60, 61). This may be explained by the alleviation of low-grade systemic inflammation, which adversely affects regulatory hormones for sleep mechanisms and is associated with disturbed sleep quality (62–64). In addition, a large body of literature supports the role of regular PA in improving sleep quality (65, 66). This is attributed to the effect of PA in improving hormonal balance, reducing inflammation, and increasing the need for longer sleep to allow muscle recovery (67, 68). In the same context, it is worth noting that the role of PA is increasingly emphasized as an influential factor for human health as revealed by recent work by Ammar et al. reporting that low PA was a considerable risk factor for the global disease burden, as low PA contributed 0.6% of all age-standardized disability-adjusted life years globally in 2019 (69).

### Sleep quality

Smoking is a detrimental factor that has been repeatedly linked to decreased sleep quality (70, 71). Similar to previous studies, we found that non-smokers had better self-evaluated sleep quality. This was consistent with extant evidence that non-smokers are less prone to developing low-grade systemic inflammation (72, 73), which is directly associated with decreased sleep quality because smoking is a leading source of potent oxidizing agents and harmful chemical toxicants that trigger an inflammatory response (73). The anti-inflammatory bioactive substances in plant foods have been shown to alleviate inflammatory conditions and minimize the risk of decreased sleep quality (25). This has been demonstrated in previous studies investigating sleep quality among adopters of the Mediterranean diet compared with those eating Western diets, with the latter having consistently poorer sleep quality than their Mediterranean diet counterparts (53, 60, 74). Therefore, the low intake of antioxidants and bioactive phytochemicals from plant foods among smokers (75, 76) may indirectly aggravate oxidative stress and subsequent inflammatory status, resulting in poor sleep quality. Similarly, excessive fat intake, particularly heat-treated commercial fat in fast foods, is known for its high proinflammatory potential (77, 78), which adversely affects sleep quality (62–64). Therefore, our finding that quality of sleep was better among participants that reduced modification in consumed delivered foods throughout RF was unsurprising.

We also found that lower screen use was significantly associated with better sleep quality among participants during RF. Exposure to short-wavelength light (blue light) that is emitted from digital devices and fluorescent/LED bulbs (79, 80) before bedtime may reduce sleepiness, increase alertness, and affect sleep quality (81). Upon exposure to blue light, retinal ganglion cells send signals to the “central body clock” (i.e., the suprachiasmatic nucleus of the hypothalamus) *via* the retinohypothalamic tract that inhibits the secretion of melatonin and stimulates the secretion of the stress hormone cortisol (82).

### Sleep disturbance

Consumption of traditional homemade foods is expected to replace frequent ordering of delivered foods, which may be associated with improved sleep quality and decreased sleep disturbance (83). In many Western diets, animal protein foods are mostly consumed in the form of high-fat foods, such as fried or processed meats (e.g., fried chicken, luncheon meat, and burgers) (84), which are associated with an elevated inflammatory state (85). However, in other parts of the world (non-Western countries), animal protein is cooked through various heat treatments (e.g., baking, braising, and stewing), which decreases the risk of potential inflammation resulting from consuming processed animal proteins (86, 87). Unexpectedly, we found that both delivered foods and traditional homemade foods were associated with an increased risk for sleep disturbance. In contrast to existing evidence (88), consumption of animal protein was not associated with high sleep disturbance or poor sleep quality in our study.

### Strengths and limitations

To our knowledge, this is the largest study pertaining to the effect of RF on dietary, sleep, and lifestyle modifications in terms of sample size during the COVID-19 period. Our sample included different populations from different countries, and from various racial and ethnic backgrounds. Therefore, this was the first multi-national study to evaluate the complex relationships between different eating behaviors, dietary intake, lifestyle behaviors, and sleep quality parameters in the context of RF on such a large scale. Furthermore, our SEM provided robust results regarding the relationships between different dietary and lifestyle factors and three distinct sleep parameters among individuals observing RF. Although this study had several strengths, we acknowledge that there were some limitations. First, the claim that SEM allows conclusions regarding causal relationships between dependent and independent variables has been critically revised (89); therefore, causality cannot be inferred in the present cross-sectional study. Second, all data were based on self-report and therefore may entail memory recall, which might have introduced recall and social desirability biases. In addition, depending on when participants completed the survey, the number of self-reported days fasted during Ramadan needed to be estimated if the survey was completed during Ramadan, and might not have been accurate because of unforeseen circumstances such as illness or travel. Moreover, the use of an online web-based survey and non-random, convenience sampling techniques might have introduced selection bias, meaning it is difficult to generalize the study results to all people fasting during RF. Although our questionnaire was derived from validated questionnaires, the lack of validation of the developed questionnaire in the present study might have resulted in some inaccuracies and inconsistencies. Fourth, the lack of a clear exclusion statement for participants diagnosed with eating and sleep disorders might have introduced some inaccuracies in the reported findings, although eating and sleep disorders are not very well diagnosed in many low- and middle-income population groups. Similarly, the lack of exclusion criteria for specific populations (e.g., athletes and older adults) might have decreased the homogeneity of the study population and allowed other factors to interfere with the targeted outcomes. Firth, the lack of control non-fasting group may allow for the effect of confounding factors to impact the sleep outcomes during RF. Finally, data were collected during lockdowns because of the COVID-19 pandemic, which might have interfered with habitual dietary and lifestyle behaviors during RF in non-pandemic times (90). This is an important factor to consider as the lockdown period was reported to induce favorable and unfavorable dietary and lifestyle behaviors, as well as certain changes in circadian rhythm, PA, and sleep quality (27, 30, 31, 39).

## Conclusion

The SEM analysis showed that consuming plant-based proteins and practicing PA were strongly correlated with optimal sleep duration (7–9 h) among the Muslims who were fasting during the Ramadan month. In addition, smoking was significantly linked to both increased sleep disruption and decreased sleep quality. Consumption of dates, vegetables, fruits, and plant-based proteins appeared to be related to better-quality sleep. Furthermore, better sleep quality was linked to the decreased use of electronic devices (i.e., less exposure to blue light) and decreased consumption of delivered foods at night during RF. Contradictory findings were discovered regarding the connection between the three sleep parameters and eating-in versus eating-out. These findings suggested that improving the intake of fruits, vegetables, and plant-based proteins during RF are important factors that could help improve sleep quality during the month of Ramadan. Regular practice of PA and avoiding smoking are also important factors that may aid in improving sleep among individuals who practice fasting during Ramadan.

## Data availability statement

The raw data supporting the conclusions of this article will be made available by the authors, without undue reservation.

## Ethics statement

The study was conducted adhering to the code of ethics of the Helsinki Guidelines. Before collecting data, the study was approved by the Social Sciences Research Ethics Committee (REC) of the United Arab Emirates University (Approval Number ERS-2021-7308) and Tehran University of Medical Science (Approval Number IR.TUMS.FNM.REC.1400.022). Furthermore, the objectives and procedures of the study were stated before seeking informed consent from participants. The patients/participants provided their written informed consent to participate in this study.

## Ramadan intermittent fasting research collaborators

National Pirogov Memorial Medical University, Vinnytsia, Ukraine: Abasi-Okot Akpan Udoyen (orcid.org/0000-0002-2947-4416). Faculty of Medicine, Al Quds University, Jerusalem, Palestine: Abdelrhman Muwafaq Janem (orcid.org/0000-0002-9854-7965). Faculty of Medicine, Helwan University, Cairo, Egypt: Abdullah Taha Zayed (orcid.org/0000-0001-7502-6342). Al-Quds University, Bethlehem, Palestine: Adriana Johny Skafi (orcid.org/0000-0001-7183-9231). Faculty of Medicine, Mansoura University Behbbit, Samannoud, Egypt: Ahmed Ashraf Elmoghazy (orcid.org/0000-0001-5313-7949). Qatar University, Mesaieed, Qatar: Ahmed Daniyal Nawaz (orcid.org/0000-0001-9424-2665). Department of Family Medicine, College of Medicine and Health Sciences, United Arab Emirates University, Al Ain, United Arab Emirates: Ahmed Juma AlKaabi. RCSI-UCD, Ayer Keroh, Malaysia: Amalin Najiha Binti Mohd Sabri. Iran Sports Medicine Research Center, Neuroscience Institute Sports Medicine Research Center, Neuroscience Institute Tehran University of Medical Sciences, Tehran, Iran: Amir Human-Hoveidaei (orcid.org/0000-0003-4607-354X). Kasr Alainy Faculty of medicine, Cairo, Egypt: Amir N. Attia (orcid.org/0000-0002-1537-3307). Kütahya Univerity of Health Sciences, kütahya, Türkiye: Ammar Mektebi. Trinity College Dublin, Dublin, Ireland: Amna Mohammed Al Zadjali. University of Tunis El Manar, Medical School of Tunis, Military Hospital of Tunis, Tunis, Tunisia: Anis Riahi (orcid.org/0000-0002-0411-983X). Department Public Health, Universitas Aufa Royhan Di Kota Padangsidimpuan, Padangsidimpuan, Indonesia: Anto Jamma Hadi (orcid.org/0000-0003-0944-5754). Orenburg state Medical University, Orenburg, Russia: Ashish Ramesh Dubey. Services Institute of Medical Sciences, Services Hospital Lahore House officer Services Institute of Medical Science, Lahore, Paksitan: Ayesha Iqbal. Lebanese university, Beirut, Lebanon: Bachar Jalal El ali. University of Aleppo, Aleppo, Syria: Bakri Yahia Roumi Jamal. Chemistry department, American University of Beirut, Beirut, Lebanon: Baraa Moujahed Hajjar. Department of Medicine, Vinnytsia National Medical University, Abuja, Nigeria: Chika Chizitelu Madekwe (orcid.org/0000-0002-5943-1636). Dentistry Programme of Mulawarman University Kerayan, Mulawarman University, Samarinda, Indonesia: Cicih Bhakti Purnamasari (orcid.org/0000-0003-1485-7817). Dentistry Programme, Mulawarman University Medical Education, Samarinda, Indonesia: Cicih Bhakti Purnamasari (orcid.org/0000-0003-1485-7817). Jordan University of Science and Technology, Irbid, Jordan: Dawlah Qasem Murshed Ahmed Saeed (orcid.org/0000-0001-8399-7923). Sbks Medical College, Ahmedabad, India: Dhaval Maunishkumar Shah (orcid.org/0000-0001-5425-8312). Public Health Asharej, Jawarneh MPH UAEU, Al Ain, United Arab Emirates: Dima Ibrahim (orcid.org/0000-0001-9394-6572). Faculty of Medicine, Dental Medicine and Pharmacy of Fez, Sidi Mohammed Ben Abdellah University, Fez, Morocco: Diyae Khadri. College of Medicine, National University for Science and Technology, Seeb, Oman: Eman Younis Al-Fahdi (orcid.org/0000-0001-5533-5914). Ambulatory Healthcare Services, Abu Dhabi, United Arab Emirates: Fatema Al Mazrouei. Department of Family Medicine, College of Medicine and Health Sciences, United Arab Emirates University, Abu Dhabi, United Arab Emirates: Fatema Al Mazrouei. Dubai Medical College, Dubai, United Arab Emirates: Fatema Muneer Radhi (orcid.org/0000-0001-5972-3543). Dubai medical college, Dubai, United Arab Emirates: Fatema Yusuf Aljanabi (orcid.org/0000-0002-6619-1829). Ambulatory Healthcare Services, Abu Dhabi, United Arab Emirates: Fatima Al sheriff Al Zaabi. Department of Family Medicine, College of Medicine and Health Sciences, United Arab Emirates University, Abu Dhabi, United Arab Emirates: Fatima Al sheriff Al Zaabi. Department of Endocrinology Syria, Faculty of Medicine, Aleppo University Hospital, University of Aleppo, Aleppo, Syria: Fatima Alzhra Mohamed Hanifa (orcid.org/0000-0003-1320-1795). Department of surgery, Jaber alahmed hospital, Kuwait City, Kuwait: Fatma Mustafa Ridha (orcid.org/0000-0002-3152-2560). Spinghar Thoracic Surgery Kabul, Kabul, Afghanistan: Fayaz Ahmad Momand (orcid.org/0000-0003-2964-8882). College of Food and Agriculture, United Arab Emirates University, Dubai, United Arab Emirates: Fayeza Hasan (orcid.org/0000-0002-8349-1057). Alexandria faculty of medicine, General practitioner, Alexandria, Egypt: Filopater Mar Gerges (orcid.org/0000-0002-3945-7417). Department of Nutrition Science, Universitas Muhammadiyah Surakarta, Sukoharjo, Indonesia: Firmansyah Firmansyah Firmansyah (orcid.org/0000-0002-9764-3461). Tanjungpura University, Pontianak, Indonesia: Frederick Putra Wijaya (orcid.org/0000-0002-4084-5134). MidHudson Family Medicine residency, Institute for family Health Family Medicine, Centerville, United States: Hassan B Nagy (orcid.org/0000-0001-9589-1333). OnDokuz Mayis University, Samsun, Turkey: Hussam Kiwan (orcid.org/0000-0003-1464-0511). Faculty of Medical Sciences, Lebanese university, Beirut, Lebanon: Ibrahim Khaled Salah El Din (orcid.org/0000-0002-3420-1279). An-Najah National University, Nablus, Palestine: Israa Hasan Hasan (orcid.org/0000-0002-4289-4802). University of Jordan, Amman, Jordan: Jehad Firas Samhouri (orcid.org/0000-0002-4878-7362). Ondokuz Mayis University, Samsun, Turkey: Kamil Sannah (orcid.org/0000-0002-8428-5191). MPH, North South University, Dhaka, Bangladesh: Lamisa Rahman (orcid.org/0000-0002-6100-6276). Aljabili Saglik Bilimleri Üniversitesi, Istanbul, Turkey: M. Munir (orcid.org/0000-0001-8104-7078). Monash University, Fawkner, Australia: Malik Bendak (orcid.org/0000-0003-3769-3974). Ain Shams General Hospital House, Khartoum, Sudan: Maram sirelkhatim elsayed (orcid.org/0000-0003-3560-9900). Tripoli central hospital, Tripoli, Libya: Marwa Mohammed morgom. Aleppo University Hospital, Aleppo, Syria: Maya Shahadeh Alassadi (orcid.org/0000-0001-9803-3422). Faculté de Médecine et de Pharmacie de Rabat, Temara, Morocco: Meryem Gounni (orcid.org/0000-0002-0360-2176). NGHA, KAMC, Riyadh, Saudi Arabia: Moath Ahmed Aldafas (orcid.org/0000-0002-9816-0672). Surgery department (Intern doctor), Princess Basma teaching hospital, Irbid, Jordan: Mohammad Mahmoud Jarrah (orcid.org/0000-0002-3339-9295). University of Aleppo, Aleppo, Syria: Mohammad / Shahrour (orcid.org/0000-0001-7506-9924). Medical Facuilty, Paktia University, Kabul, Afghanistan: Mohammad Elyas Wardak (orcid.org/0000-0002-7584-991X). Student Research Committee, Iran Clinical Research Development Center of Imam Khomeini Hospital, Jiroft University of Medical Sciences, Jiroft, Iran: Mohammad Pourfridoni (orcid.org/0000-0002-0510-3194). College of Medicine, Qatar University, Doha, Qatar: Mohammad Zulqurnain Haider (orcid.org/0000-0003-0598-5171). Faculté de Médecine, de Pharmacie et de Médecine Dentaire de Fès, Fez, Morocco: Mohammed Chakir (orcid.org/0000-0001-5232-4435). Kas al aini Clinic, Cairo, Egypt: Mohammed Al-Rsheed mostafa Omar Abueissa (orcid.org/0000-0002-8908-9026). Department of Physiology, Alzaiem Alazhari University, Khartoum, North Sudan: Mohannad Abdalfdeeel Almahie Shaban (orcid.org/0000-0002-2670-4802). Department of Human Physiology, Alzaiem Alazhari University, Khartoum, North Sudan: Mohannad Abdalfdeel Almahie Shaban (orcid.org/0000-0002-2670-4802). Ministry of Health Internship, Khartoum, Sudan: Monzir Musa Hamdan Mohammed (orcid.org/0000-0001-6385-8117). Ministry of Health, Khartoum, Sudan: Mosab Salah elmahi Ahmed (orcid.org/0000-0003-1056-1402). The Department of Statistics, The Islamia University of Bahawalpur, Bahawalpur, Pakistan: Muhammad Daniyal Khan (orcid.org/0000-0002-1415-4970). College of Medicine, Menoufia University, Al Minufiyah, Egypt: Muhammad Sameh Amer (orcid.org/0000-0002-6562-3319). Psychology Department of Behavioral Science, Rehman College of Dentistry, Peshawar, Pakistan: Muttahid Shah (orcid.org/0000-0003-3423-9549). CMHS Family Medicine, UAEU, Al Ain, United Arab Emirates: Nadirah Ghenimi Ghenimi (orcid.org/0000-0003-0897-2587). Healthcare Psychology, Abu Dhabi, United Arab Emirates: Nailah Mahmood (orcid.org/0000-0001-5834-9422). ICU department, Alexandria Main University Hospital, Alexandria, Egypt: Nermeen Mohammed Afifi (orcid.org/0000-0001-5614-3340). Mosul medical collage, Mosul, Iraq: Noran Omar Mahmood. Ondokuz Mayis University, Samsun, Turkey: Noura Ahmad Kanjo (orcid.org/0000-0002-2348-1156). Emirates Health Services, Dubai, United Arab Emirates: Rahaf Ziad Abughosh (orcid.org/0000-0003-3225-6927). Faculty of Medicine, Ain Shams University, Cairo, Egypt: Ramy Rafaat Yassa (orcid.org/0000-0002-0675-9320). Department of Nutrition, Pertamedika College of Health Sciences, Jakarta, Indonesia: Rani Rahmasari Tanuwijaya M. Gizi (orcid.org/0000-0003-3438-0614). School of Nursing and Midwifery, Tehran University of Medical Sciences, Tarbiat Modares University, Tehran, Iran: Reza Heidari-Soureshjani (orcid.org/0000-0002-1212-1171). Shadan Institute of Medical Sciences and Research Centre, Peeramcheru, India: Romana Riyaz (orcid.org/0000-0003-0113-9824). CMH Institute of Medical Sciences, Multan, Pakistan: Rutab Tareen (orcid.org/0000-0002-1215-7991). Birat Medical College and Teaching Hospital Sukhrampur, Krishnanagar, Nepal: Sajjad Ahmed Khan (orcid.org/0000-0002-5315-9934). Kuwait University, Kuwait City, Kuwait: Sana Kalim Qureshi (orcid.org/0000-0001-7640-8334). Jordan University of Science and Technology, Irbid, Jordan: Sara Mohammed Ahmed Musleh Al-Badani (orcid.org/0000-0002-6986-8647). Alexandria Faculty of Medicine, Alexandria, Egypt: Sara Nazmy Ataallah (orcid.org/0000-0002-5900-1361). College of Medicine, King Saud bin Abdulaziz University for Health Sciences, Riyadh, Saudi Arabia: Saud Mohammed Alwatban (orcid.org/0000-0003-4787-7486). King Abdulaziz Medical City, Ministry National Guard Health Affairs, Riyadh, Saudi Arabia: Saud Mohammed Alwatban (orcid.org/0000-0003-4787-7486). King Abdullah International Medical Research Center, Riyadh, Saudi Arabia: Saud Mohammed Alwatban (orcid.org/0000-0003-4787-7486). Nangarhar Medical Faculty, Timergara, Pakistan: Sayed Mustafa Kamal (orcid.org/0000-0002-7098-8443). Khatam-Al-Nabieen University, Kabul, Afghanistan: Shams Ul Haq n/a Noori (orcid.org/0000-0002-8249-2023). University of Debrecen, Debrecen, Hungary: Somto Judith Okafor (orcid.org/0000-0001-9455-2348). Faculté de médecine d’Alger, Algiers, Algeria: Tadjadit Lydia (orcid.org/0000-0002-0294-3438). Sultan Qaboos University (Oman), Seeb, Oman: Tariq Ali Al Habsi (orcid.org/0000-0001-9010-8856). JSS Medical College, Mysore General Medicine, Kannur, India: Tejaswini Ashok (orcid.org/0000-0002-5888-0106). Jimma University, Jimma, Ethiopia: Tujuba Diribsa Benti (orcid.org/0000-0001-8453-5755). DNB GEM Hospital General Medicine, Chennai, India: Waseem N Ahmed (orcid.org/0000-0002-1687-922X). An-Najah National University, Bethlehem, Palestine: Yazan William Giacaman (orcid.org/0000-0002-3277-2481). MWACP West African College of Physicians, Federal Neuropsychiatric Hospital, Maiduguri, Nigeria: Yesiru Adeyemi KAREEM (orcid.org/0000-0001-5569-6592). MBBCh Dubai Medical College, Manama, Bahrain: Zainab Sadeq AlRabeea (orcid.org/0000-0002-8656-4557).

## Author contributions

All authors provide substantial contributions to the conception or design of the work or the acquisition, analysis, or interpretation of data for the work, drafting the work or revising it critically for important intellectual content. The collaborators were involved in collecting the data, revising the manuscript critically, and finalizing and approving the final version.
