# Supplementary material for: Examination of sleep in relation to dietary and lifestyle behaviors during Ramadan: A multi-national study using structural equation modeling among 24,500 adults amid COVID-19
Source: Front Nutr. 2023 Mar 8;10:1040355. doi: 10.3389/fnut.2023.1040355 (PMC10030961; doi:10.3389/fnut.2023.1040355)
Supplement: Supplementary file 1 [file Table_1.DOCX]

**Supplementary Table 1.** Discriminant validity of the study variables (N=24,541)

|  | 1 | 2 | 3 | 4 | 5 | 6 | 7 | 8 | 9 | 10 | 11 | 12 | 13 | 14 | 15 |
| --- | --- | --- | --- | --- | --- | --- | --- | --- | --- | --- | --- | --- | --- | --- | --- |
| 1. Smoking | - |  |  |  |  |  |  |  |  |  |  |  |  |  |  |
| 2. Food delivered | 0.07 | - |  |  |  |  |  |  |  |  |  |  |  |  |  |
| 3. Restaurant dining | 0.09 | 0.43 | - |  |  |  |  |  |  |  |  |  |  |  |  |
| 4. Computer use | **0.01** | 0.05 | **0.02** | - |  |  |  |  |  |  |  |  |  |  |  |
| 5. Physical activity | 0.05 | **0.001** | **0.03** | **0.03** | - |  |  |  |  |  |  |  |  |  |  |
| 6. Vegetables, fruits, and dates consumption | 0.05 | **0.03** | **0.02** | 0.10 | **0.02** | - |  |  |  |  |  |  |  |  |  |
| 7. Cereals, pulses (dried legumes), and bakery products consumption (plant-protein sources) | **0.001** | 0.05 | 0.05 | 0.07 | **0.04** | 0.24 | - |  |  |  |  |  |  |  |  |
| 8. Milk, fish, chicken, and meat consumption | **0.04** | **0.03** | **0.02** | 0.11 | 0.01 | 0.32 | 0.32 | - |  |  |  |  |  |  |  |
| 9. Oils, fats, and fried foods consumption | **0.001** | 0.10 | 0.06 | 0.07 | 0.11 | 0.15 | 0.35 | 0.26 | - |  |  |  |  |  |  |
| 10. Beverages | 0.05 | 0.11 | 0.08 | 0.11 | 0.05 | 0.12 | 0.30 | 0.19 | 0.34 | - |  |  |  |  |  |
| 11. Salt and salty snacks consumption | **0.02** | 0.07 | 0.05 | 0.10 | 0.09 | 0.16 | 0.27 | 0.22 | 0.39 | 0.42 | - |  |  |  |  |
| 12. Homemade traditional foods consumption | 0.05 | **0.001** | **0.01** | 0.15 | **0.04** | 0.27 | 0.26 | 0.29 | 0.25 | 0.23 | 0.33 | - |  |  |  |
| 13. Sleep duration | **0.01** | **0.02** | **0.001** | **0.001** | 0.05 | **0.02** | **0.04** | **0.02** | **0.001** | **0.01** | **0.001** | **0.001** | - |  |  |
| 14. Self-described sleep quality | **0.02** | **0.02** | **0.001** | 0.09 | 0.08 | **0.04** | **0.02** | **0.001** | **0.03** | **0.01** | **0.04** | **0.03** | 0.28 | - |  |
| 15. Sleep disturbance | **0.04** | **0.02** | **0.02** | 0.05 | **0.03** | 0.11 | 0.06 | 0.08 | **0.02** | **0.01** | **0.02** | 0.08 | 0.12 | 0.30 | - |
